# Small Bowel Imaging from Stepchild of Roentgenology to MR Enterography: Part I: Guidance in Performing and Observing Normal and Abnormal Imaging Findings

**DOI:** 10.3390/life13081691

**Published:** 2023-08-05

**Authors:** Antonio Pierro, Laura Maria Minordi, Luigi Larosa, Giulia Guerri, Alessandro Grimaldi, Fabio Quinto, Fabio Rotondi, Annalisa Marcellino, Teresa Bevere, Raffaella Basilico, Roberto Iezzi, Savino Cilla

**Affiliations:** 1 Radiology Unit, San Timoteo Hospital, 86039 Termoli, Italy; antonio.pierro@asrem.org (A.P.); teresa.bevere@asrem.org (T.B.); 2 Radiology Unit, Fondazione Policlinico Universitario Agostino Gemelli IRCCS, 00168 Roma, Italy; lauramaria.minordi@policlinicogemelli.it (L.M.M.); luigi.larosa@policlinicogemelli.it (L.L.); giulia.guerri@policlinicogemelli.it (G.G.); alessandro.grimaldi@policlinicogemelli.it (A.G.); roberto.iezzi@policlinicogemelli.it (R.I.); 3 Angiography Unit, “L. Bonomo” Hospital, 70031 Andria, Italy; quintofabio@libero.it; 4 Oncological Surgery Unit, Gemelli Molise Hospital, 86100 Campobasso, Italy; fabio.rotondi@gemellimolise.it; 5 ASReM, 86100 Campobasso, Italy; annalisa.marcellino@asrem.org; 6 Department of Neurosciences, Imaging and Clinical Studies, “Gabriele D’Annunzio” University, 66100 Chiety, Italy; raffaella.basilico@asl2abruzzo.it; 7 Medical Physics Unit, Responsible Research Hospital, 86100 Campobasso, Italy

**Keywords:** small bowel, MR enterography, Crohn’s disease, bowel wall enhancement patterns

## Abstract

MRE has become a standard imaging test for evaluating patients with small bowel pathology, but the indications, interpretation of imaging findings, methodology, and appropriate use must be standardized and widely known. Several signs of small bowel damage in inflammatory and non-inflammatory small bowel pathology include strictures, abscess, inflammatory activity, sinus tract, wall edema, fistula, mucosal lesions, and mesentery fat hypertrophy, all of which are widely and accurately explained by MRE. MRE is a non-invasive modality that accurately assesses the intra-luminal, parietal, and extra-luminal small bowel. The differential MRE appearance allows us to distinguish between different small bowel pathologies, such as neoplastic and non-neoplastic small bowel diseases. The purpose of this paper is to present the MRE technique, as well as the interpretation of imaging findings, through the approach of a rigorous stepwise methodology.

## 1. History Overview of Small Bowel Imaging

Referred to as the “Stepchild of Roentgenology” by Richard Schatzki [1], the X-ray demonstration of small bowel disease was poorly investigated until 1940. Radiographic study of the small intestine was challenging due to rapid peristalsis and overlapping bowel loops. An adequate examination of the small intestine became possible only after the feasibility of barium supplied through a duodenal tube. In 1929, Gilberto Pasquera of New York was the first to use the duodenal tube for continuously controlled filling of the small intestine. Ten years later, Jacob Gershon-Cohen introduced enteroclysis, which allowed the successful evaluation and characterization of small bowel pathology. Unfortunately, due to the intrinsic limits of the method, studies using barium had a limited role in diagnosing acute small bowel obstruction or ileus and assessing extraluminal disease. The main drawbacks of conventional enteroclysis, including the lack of direct information on the state of the bowel wall and the extramural extension, compromised its efficacy [1]. Although barium examinations remain a formidable tool in the depiction of mucosal abnormalities, particularly the apthoid lesions of early Crohn disease [2], magnetic resonance enterography (MRE) possesses the same significant advantages, namely, the delineation of extraintestinal and transmural inflammation, multiplanar evaluation, and high intrinsic soft-tissue resolution, without radiation exposure [3]. In particular, young Crohn’s patients, who require appropriate diagnostic and surveillance imaging at diagnosis and during symptomatic and asymptomatic recurrence, profit from this marginally invasive method [4].

Nowadays, the traditional radiological techniques, such as enteroclysis, are increasingly being replaced by contemporary endoscopic and imaging techniques, namely, computed tomography (CT) and MRE [5,6]; in particular, magnetic resonance imaging (MRI), due to the lack of ionizing radiation and good tissue contrast resolution, has gained popularity as a tool for assessing and diagnosing small bowel disease [7].

In recent years, capsule endoscopy, also known as wireless capsule endoscopy or video capsule endoscopy, has gained considerable interest in evaluating the gastrointestinal tract when traditional endoscopic techniques have failed [8]. In particular, it allows for more sensitive intestinal mucosa evaluation than radiological procedures. Despite having good patient tolerability and safety profiles, its main limitation is that stenotic disease and extraluminal penetrating complications may not be assessed. Cross-sectional imaging, on the other hand, such as MRE, allows for the evaluation of transmural lesions, as well as evaluates stricturing and penetrating complications [9].

## 2. Macroscopic Anatomy Features of Small Bowel

**Figure 1:**
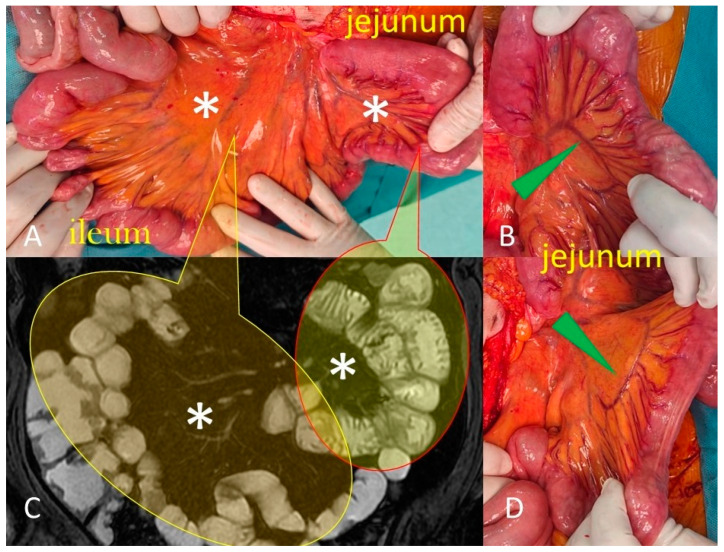
Surgical images (**A**,**B**,**D**) show that compared to the ileum, the jejunum has less fatty mesentery (asterisk in **A**,**C**) and longer and straighter vasa recta (green arrowheads in **B**,**D**).

The small intestine is the largest organ in the gastrointestinal (GI) system. It controls nutrient absorption, water and electrolyte balance, immunity, and endocrine secretion [10]. The small intestine is a hollow tube that starts at the pylorus and ends at the ileocecal valve, measuring 6 to 7 m in length [11]. The duodenum, jejunum, and ileum are the three components of the small bowel. Because of its retroperitoneal location, immobility, and lack of a mesentery, the duodenum is frequently considered a distinct anatomical entity. Due to their relationship to a suspensory mesentery, the jejunum and ileum have considerable mobility within the abdominal cavity [12]. The term “small bowel” (small intestine) usually refers to the jejunum and ileum only, excluding the duodenum [12]. The free margin of the small intestinal mesentery encases the jejunum and ileum, allowing them to move around freely within the abdominal cavity. The mesentery of the small intestine is a double-layered peritoneum sheet that is fan-shaped and attached to the posterior abdominal wall along the root of the small-bowel mesentery (SBM) [12]. The SBM is a voluminous, fat-laden peritoneal reflection that fixes the jejunum and ileum to the posterior abdominal wall. The associated parietal boundary is approximately 15 cm long and goes obliquely down from the duodenojejunal flexure to the ileocecal area [13]. The superior mesenteric artery (SMA) and superior mesenteric vein (SMV) run through the root of the SBM. The small intestine mesentery, as well as the entire length of the jejunum and ileum, are entirely located in the infracolic compartment of the peritoneal cavity, beneath the attachment of the transverse mesocolon [12]. There is no distinct anatomic feature that defines the transition from the end of the jejunum to the beginning of the ileum; the jejunum is centrally placed in the abdomen, whereas the ileum is mostly located in the hypogastric area and pelvic cavity [10]. More precisely, The jejunal loops are usually seen in the top left corner of the infracolic compartment. In contrast, the loops of the ileum tend to be found in the lower part of the abdominal cavity, mainly on the right side, and often flopped into the pelvic cavity [10]. The jejunum has a thicker mucosal lining, a thicker wall, a greater diameter, a less fatty mesentery, and a longer and straighter vasa rectum than the ileum [8,10] (Figure 1).

**Figure 2:**
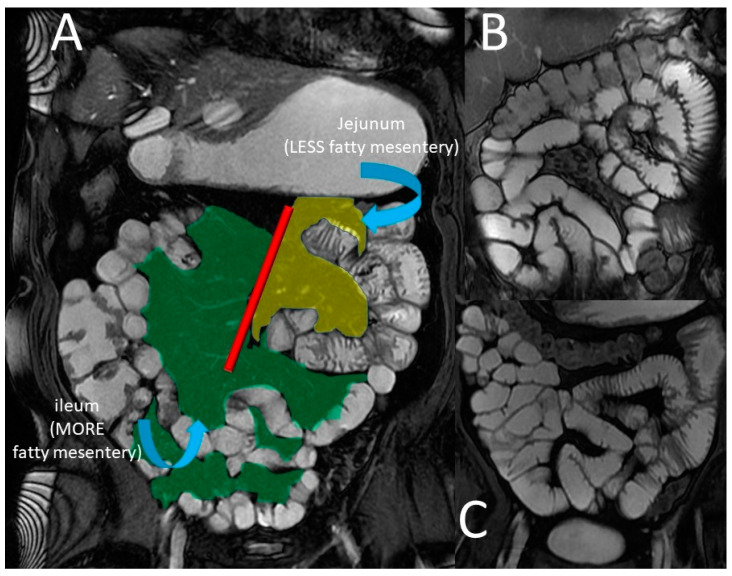
Normal MRE features of small bowel–coronal gradient echo sequences (FIESTA) (**A**): Compared to the ileum, the jejunum has less fatty mesentery (**A**) and more valvulae conniventes (**B**,**C**); yellow area, mesentery of the jejunum; green area, mesentery of the ilium. Red line, root of the mesentery.

**Figure 3:**
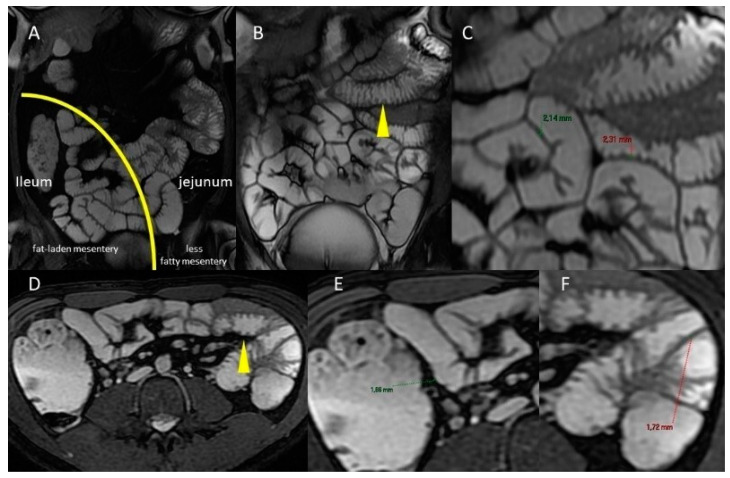
Normal MRE features of small bowel–coronal gradient echo sequences (FIESTA) (**A**) Compared to the ileum, the jejunum has less fatty mesentery and prominent valvulae conniventes. In (**A**), an imaginary curved line with a left convexity divides the abdomen in the region hosting the jejunum from that hosting the ileus. Coronal (**B**) and axial (**D**) FIESTA sequences (**B**): jejunum prominent valvulae conniventes (yellow arrowhead). Coronal (**C**) and axial (**E**,**F**) FIESTA sequences: The wall is normally <3 mm thick.

Moreover, the jejunum is characterized by valvulae conniventes (circular mucosal and submucosa folds that serve to increase surface area) [12] that are more prominent in the proximal intestine and decrease throughout the small intestine [8] (Figure 2 and Figure 3).

**Figure 4:**
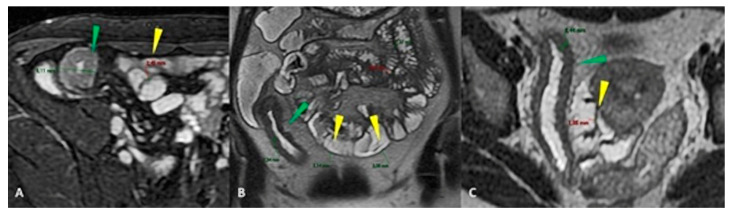
Axial (**A**) FIESTA sequence: Wall thickening of 9mm (green arrowhead). Coronal T2-weighted (**B**,**C**) wall thickness (green arrowhead in **B**,**C**). Normal wall thickness <3 mm (yellow arrowhead in **A**–**C**).

**Figure 5:**
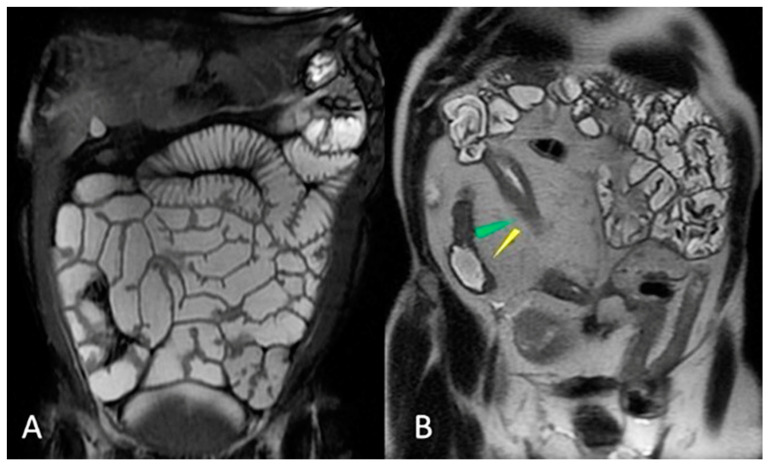
Coronal (**A**) FIESTA sequences do not show fibrofatty proliferation of normal mesenteric fat with kissing bowel loops. Coronal T2-weighted images (**B**) show fibrofatty proliferation (creeping fat), i.e., hypertrophy of the mesenteric fat, which separates bowel loops as a sign of mesenteric inflammation in Crohn’s disease. Affected bowel loops are separated by focal/regionally increased fat (fibrofatty proliferation or creeping fat): Opposed green and yellow arrowheads in (**B**).

The “terminal ileum” refers to the last 10 to 20 cm of the ileum, which is a more common site of small bowel pathology. The small bowel’s usual intraluminal diameter is 2.5 to 3.0 cm and decreases throughout its length. The typical thickness of the wall is 1 to 2 mm (Figure 3). The fibrofatty proliferation, i.e., the hypertrophy of the mesenteric fat, is not observable in the mesentery of a healthy small intestine (Figure 4 and Figure 5).

Therefore, the healthy intestinal loops are not spaced apart from each other enough to touch with their serosa (“kissing bowel loops”).

## 3. Guidance in Performing MRE

In MR, an adequate distension is indispensable for reliable small bowel assessment. This can be achieved in two ways: enterography (oral administration) or enteroclysis (nasojejunal intubation with the tube tip close to the ligament of Treitz) [14]. It is shown that enteroclysis allows a better loop distension and visualization of mucosal abnormalities in comparison to enterography [15]. Nevertheless, enterography does not decrease diagnostic accuracy, and it is a less invasive and faster examination.

In 2013, The European Crohn’s and Colitis Organization (ECCO) and the European Society of Gastrointestinal and Abdominal Radiology (ESGAR) released an evidence-based consensus for small bowel assessment [16]. The guidelines specifically recommended using MRI fast sequences, which acquire T1- and T2-weighted images within a single breath hold while reducing motion and peristaltic artifacts.

The use of the intravenous injection of contrast medium is mandatory and currently recommended for assessing bowel wall enhancement patterns and mesenteric vessels unless contraindicated [17]. The inclusion of small bowel motility evaluation via cine images may improve the lesion detection rate over static MRE alone [17].

In particular, reduced peristalsis is visible in small bowel areas of active fibrosis, inflammation, or in a neoplastic loop; therefore, the appearance of reduced peristalsis provides more diagnostic confidence in classifying a loop of the small intestine as pathological [17,18,19]. Biphasic contrast agents, including many non-absorbable iso-osmolar solutions (poly-ethylene glycol or mannitol solutions), are required to achieve a negative effect on T1-weighted images and a positive effect on T2-weighted images (“water-like” effect), also providing a suitable small bowel dilation without the side effects [16]. Among the various enteric contrast agents, the biphasic category is the most common. On T1-weighted images, the “black lumen” is critical to assess the bowel mucosa and to detect mural enhancement after intra-venous contrast (IVC) treatment [15].

Common clinical protocols require and the expert consensus recommends that the patient must fast for 4 or 6 h before the MRE procedure [20]; a greater tolerance for high-volume oral contrast ingestion is obtained with an empty, non-dilated stomach [21]. Fasting reduces the number of food remnants and debris in the intestinal lumen, which might be misinterpreted for polyps or mass lesions. Patients must also follow a low-residue diet unless contraindicated for the previous five days. Because feces can cause delays in small bowel transit times, a low-residue diet stimulates the decrease of fecal matter in the colon, which facilitates the migration of the small-bowel contrast agent. [15,17,22]. The patients do not need to follow a colon preparation regimen prior to MRE [17,23]. Nonetheless, cathartics may be helpful in individuals with suspected colonic and rectal illness, and diffusion-weighted imaging (DWI) should be used to increase diagnostic accuracy in patients with unclean intestines [17].

The expert consensus recommends fasting for at least 4 h before performing MRE [24]. A few authors recommend carrying out a preparation with laxatives before carrying out the MRI examination [25]. The amount of oral contrast material that patients must ingest varies between 1000 and 2000 mL; however, patient tolerance, size, history of bowel resection, and the existence of an ileostomy can all influence the result [15,17,23].

Using polyethylene glycol (PEG), The typical time for the contrast column to reach the cecum ranges between 55 and 65 min. [15]. Patients should drink a glass of the preparation every 3 min, under the monitoring of a nurse, avoiding any not tolerated overload that could lead to vomiting, thus, precluding the examination. Immediately before imaging, patients should drink an additional 250–500 mL of water, depending on tolerance [15,17,23].

The lack of intestinal wall motion on acquired images is a crucial requirement for the diagnostic assessment. The stimulating impact of ingested oral contrast material on the bowel results in increased peristalsis, which might result in motion artifacts and equivocal interpretations. Approximately 80% of institutions administer a hypoperistaltic medication before or during MRE [23], despite differences in agent, dosage, and time of delivery. Glucagon or hyoscine butylbromide (Buscopan) are the commonly used agents [23].

In many institutions, IVC administration is widely preferred over intra-muscular (IM) administration; doses are typically 0.5 to 1 mg for glucagon and 20 to 40 mg for hyoscine butylbromide [17,23]. The investigation should begin with a coronal panoramic assessment of the full intestine utilizing cine-image multiphase BSSFP or SSFSE sequences during breath-holding or free breathing before the IV injection of glucagon or hyoscine butylbromide. The multiphasic cine imaging provides functional information about bowel motility (altered motility, with reduced peristalsis movements), strictures, and adhesions. The bowel motility evaluation has increased pathologic lesion detection [18]. bSSFP imaging (TrueFISP, FIESTA, balanced FFE) is the most widely utilized technique in cine MRE [18]. The altered motility is an alarm for radiologists able to unmask the locations of potential diseases, improving confidence in the diagnosis.

Antiperistalsis drugs should be administered after the cine motility sequences; approximately 1 min later, a preliminary evaluation without contrast on the coronal plane should be obtained, followed by a dynamic T1-weighted, fat-suppressed, spoiled gradient echo in the coronal plane during breath holding with three or more phases (arterial, portal, and late phases) [17]. An axial acquisition should also be carried out after the dynamic coronal acquisition to check the bowel in a different plane. The IVC contrast enhancement is capable of demonstrating the bowel wall thickening with various patterns according to inflammatory, neoplastic, or ischemic bowel disease [15]. Subsequently, the T2-weighted sequence (with and without fat suppression) must be obtained in order to evaluate the bowel wall signal, thickness, intramural edema, and the subtle inner luminal irregularities associated with ulcerations [17,26]. In particular, it is preferable to obtain axial and coronal T2 FSE without fat suppression, axial or coronal T2 FSE with fat suppression, and axial and coronal SSFPGE without fat suppression. All scans must include the small bowel and colon from the diaphragm to perineum [26].

Diffusion-weighted imaging was also suggested [27], but it is not mandatory. Nonetheless, we strongly recommend its use because DWI improves the diagnostic accuracy of MRE [28,29,30]. DWI should be acquired in the axial plane during free breathing and must include b values ranging from 0 to 900 [26]. Restricted diffusion is associated with severe active inflammation in Crohn’s disease, but false positives are not infrequent, and the DWI results need to be correlated to the ones obtained with the usually recommended sequences [17]. Finally, it would be useful to obtain a sagittal SSFPGE scan without fat suppression to explore the pathological loops in a different plane.

Maximal slice thicknesses are suggested to be 4–5 mm for T2-weighted imaging and 3–4 mm after IVC administration in the best plane, while in cine sequences, the slice thickness is typically approximately 6–10 mm [15,17,26].

In terms of lesion detection and morphologic assessment, the prone and supine positions are comparable [31] (Moreover, imaging in the prone position reduces respiratory motion artifacts and improves luminal distension, shortening the time needed to obtain the coronal sequence by compressing the abdomen, and it results in more comfort for claustrophobic patients. Small-bowel distension of both healthy and diseased loops is statistically superior when imaging in the prone position. Therefore, patients may be examined in either position without compromising diagnostic accuracy depending on the factors that influence the choice or suggest necessity [31,32].

## 4. MRE Clinical Indications

Considering the clinical indications, MRE has become a widely acknowledged approach for evaluating the small bowel in Crohn’s disease patients, particularly in young subjects who will have to undergo repeated follow-ups with multiple imaging examinations during their lifetime to monitor treatment. However, it must be underlined that according to the existing ECCO guidelines [33], MRE is the appropriate tool in established Crohn’s disease because it allows the investigation of the extraluminal involvement. On the contrary, in suspected Crohn’s disease, the diagnostic yield of CE was demonstrated superior [33].

MRE is an established imaging approach often used to evaluate bowel disorders and, therefore, both inflammatory and non-inflammatory pathology [32].

MRE is a formidable tool for Crohn’s disease burden evaluation and a robust tool for detecting a broad spectrum of less well-known, non-inflammatory bowel and mesenteric diseases. The small intestine is not an entire field of Crohn’s disease. Small Bowel non-IBD conditions represent the other large field of application of the MRE and include pathologies of different etiologies. Although in many of these non-inflammatory pathologies, the symptoms may be similar, such as anemia, symptoms of acute or subacute small bowel obstruction, abdominal pain, or weight loss, they have a different nature and a different pathogenesis.

In this scenario, the radiologist must be able to recognize the signs of many different pathologies that affect the small intestine.

These are lymphoma, small bowel adenocarcinoma, neuroendocrine tumor, gastrointestinal stromal tumor, Peutz–Jeghers syndrome, radiation enteritis, familial Mediterranean fever, intussusception, Meckel’s diverticulum, Cowden disease, small bowel sarcoidosis, small bowel diaphragm disease, and abdominal cocoon syndrome [34,35].

In addition to Crohn’s disease, radiologists should know about uncommon small bowel disorders because they could be recognized in patients referred for small bowel MRE and simulate Crohn’s disease [36].

## 5. Small Bowel Loop Pathology on MRE

### 5.1. How to Properly Compose the Diagnostic Puzzle

Regardless of the type of small bowel pathology, a systematic approach is essential in evaluating what we achieve with small bowel imaging methods. This strategy is necessary to direct image interpretation and support the creation of a precise and concise differential diagnosis.

Despite the small pathological bowel loop that may exhibit wall thickening, intramural edema, segmental mural hyperenhancement, and stricture, these signs may vary in different diseases [28]. The contribution that each sign can express in the various pathologies can be profoundly different; therefore, radiologists must be able to compose the mosaic to formulate an accurate diagnosis [37].

Cronin et al. [38] presented normal small bowel parameters on MRE with a notable benefit to each radiologist in their everyday assessments of the small bowel disorder examination. Table 1 reports a summary of normal small bowel mean diameter on MRE at the jejunum, proximal ileum, distal ileum, and terminal ileum.

**Figure 6:**
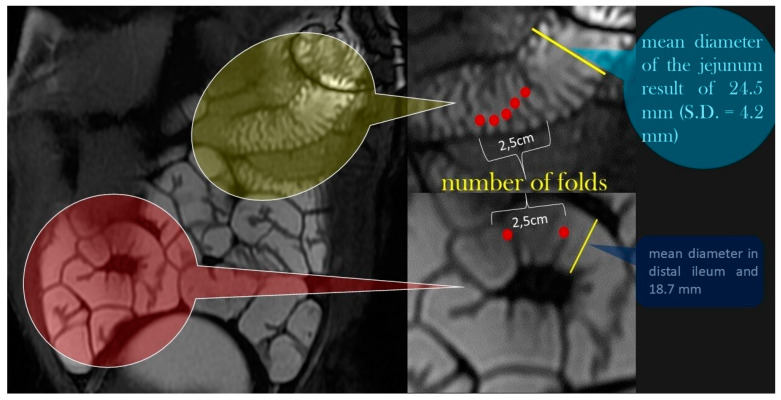
Normal small bowel parameters on MR enterography: The number of folds per 2.5 cm varied from 4.6 in the jejunum to 1.5 in the terminal ileum [15]. Folds: red spheres. Normal small bowel diameter: yellow line [15].

The number of folds per 2.5 cm varied from 4.6 in the jejunum to 1.5 in the terminal ileum. The fold thickness varied from 2.1 mm in the duodenum to 1.8 mm in the terminal ileum. In other words, the small bowel parameters gradually decreased in size from the jejunum to the smallest measurements, which were in the terminal ileum (Figure 6).

When the wall thickness is more than 3 mm, despite adequate luminal distention, an abnormal small-bowel loop is found [39]. Although submucosal edema can be accurately assessed using T2-weighted imaging, bowel wall thickening findings in MRE are broadly comparable to CT [26].

In addition, if thickening of the bowel wall is recognized, some imaging features should be considered to reduce the differential diagnosis. They are: length of involvement, level of thickness, symmetric versus asymmetric involvement, location of the lesion along the small bowel’s pathway (proximal or distal), site of the lesion (mucosal, sub mucosal, or serosal) in the small bowel wall, the pattern of enhancement, continuous involvement or discontinuous, skip lesions, and anomalies of the perienteric tissue [33,39].

### 5.2. Stepwise Methodology

The initial step is to determine the amount of bowel that is impacted. According to Macari et al. [39], pathologic conditions tend to cause focal involvement (≤5 cm), segmental involvement (6–40 cm), or diffuse involvement (>40 cm). This is not a mere speculative exercise but represents a helpful tool to narrow the field of differential diagnosis. In fact, focal alterations are more likely the expression of malignant pathologies where very segmental or extensive lesions represent benign pathologies [27,39], except for a small bowel lymphoma, which typically shows as a segmental distribution.

The second major step is the evaluation of the degree of thickening of the abnormal small bowel. As reported in Table 2, mural thickening can be stratified into three categories: mild, moderate, and marked, even though the most common disorder affecting the small intestine causes moderate to marked thickening [36].

Crohn’s disease, intestinal ischemia, intramural hemorrhage, angioedema, vasculitis, lymphoma, adenocarcinomas, and other neoplasms may cause moderate to severe small bowel thickening even though neoplasms, and sometimes, intramural bleeding, are the most common causes of small-bowel wall thickening exceeding 20 mm [27,39].

Greater than 15 mm bowel wall thickening is unusual for Crohn’s disease, and it is much more consistent with neoplasm, especially if it is asymmetric or mass-like [40].

Exploring symmetric versus asymmetric (different degrees of eccentric thickening around the circumference) wall thickening patterns is a significant landmark. In this scenario, symmetric thickening along the circumference of the small bowel suggests a benign condition, while neoplasms are more prone to an asymmetric pattern [39].

An exception to this rule is the granulomatous conditions and Crohn’s disease, which can express the asymmetric thickening or symmetric thickening in any case of small bowel lymphoma [27,39].

The third step is the evaluation of bowel wall enhancement following intravenous contrast injection.

The mucosal layer expresses the most pronounced uptake. On the other hand, the less vascular submucosa is rarely visible as a separate entity unless affected by edema, fat infiltrates, or hemorrhagic conditions. Although it is not as noticeable as the mucosa’s enhancement, the muscularis propria and serosa also exhibit contrast enhancement [41].

**Figure 7:**
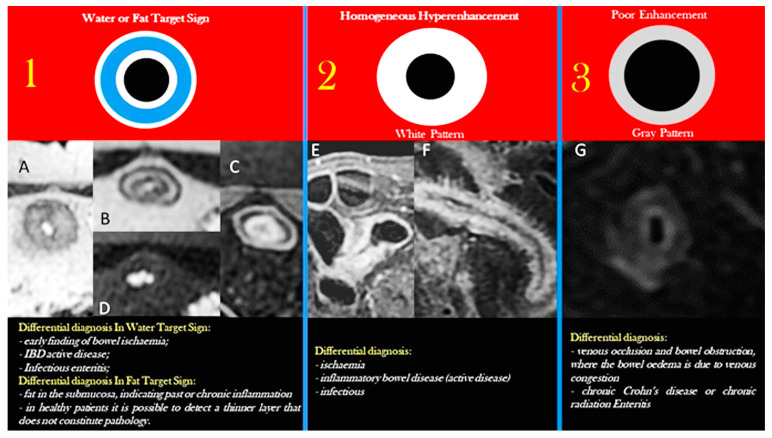
This scheme was borrowed from the enhancement patterns observable in CT enterography after the administration of an intravenous contrast medium: (**1**) target sign: indicates inflammation or ischemia of the bowel where the inner and outer high-enhancement layers correspond to the hyperemic mucosa and serosa, respectively, while the poor enhancement central layer represents the edematous or fat submucosa. (**2**) Homogeneous hyperenhancement or white pattern: global wall intense enhancement equal to or greater than that of venous vessels. (**3**) poor enhancement of the bowel wall is considered when the bowel wall is enhanced to be similar to that of the muscle. In **1A**: the axial T2-weighted MRE image shows small-bowel wall thickening, mural edema (hyperintense mural signal intensity), and luminal narrowing (active Crohn’s disease phase). **1B**,**1D**: the axial T2-weighted MRE image (**1B**) and fat-suppressed T2-weighted image (**1D**) show small bowel poor wall thickening and submucosa fat (hypointense mural signal intensity in fat-suppressed T2-weighted image) in the chronic Crohn’s disease phase. (**1C**) Axial contrast-enhanced fat-suppressed T1-weighted show stratified mural hyperenhancement. (**2**): coronal contrast-enhanced fat-suppressed T1-weighted MRE images (**2E**,**F**) show global small-bowel mural hyperenhancement. (**3**): poor enhancement. **3G**: Crohn’s disease with active inflammation; axial contrast-enhanced fat-suppressed T1-weighted images show the resolution of the mural edema and hyperenhancement.

There are four main bowel wall enhancement patterns, and they constitute a further backbone in the evaluation of small intestine pathology [39,41] (Figure 7): stratification, two (double halo sign) or three (the target appearance); homogeneous or hyperenhancement; lower enhancement. Stratified contrast enhancement subtends a benign condition of the small-bowel wall, i.e., Crohn’s disease or benign non-Crohn’s disease [42]. Stratified contrast enhancement comprises three rings of different tissue MR signals. Two high-intensity rings, i.e., mucosa (inner ring) and muscularis propria (outer ring) are separated by a low-intensity ring, which refers to the submucosa (middle ring), reflecting edema or fat composition [43]. Homogeneous mural hyperenhancement represents a global transmural high enhancement that uniformly compromises the entire bowel wall: this pattern can be present not only in active Crohn’s disease but also in other disorders such as fibrosis, infiltration, ischemia, collagen deposition, or shock bowel [40]. Less avid mural enhancement is more typical of fibro-stenotic disease [44] or in advanced-stage ischemia of the small bowel, whereas heterogeneous enhancement is most frequent with adenocarcinoma and malignant gastrointestinal stromal tumors (GISTs) [45]. The mesentery’s health and the size, location, and attenuation of lymph nodes all have a significant influence in restricting the hypothesis for a diagnosis of an abnormal small-bowel pattern. If there is an abnormal small-bowel loop, it is always mandatory to check the superior mesenteric vein and artery [39].

Table 3 reports the main enhancement patterns of the bowel wall.

**Figure 8:**
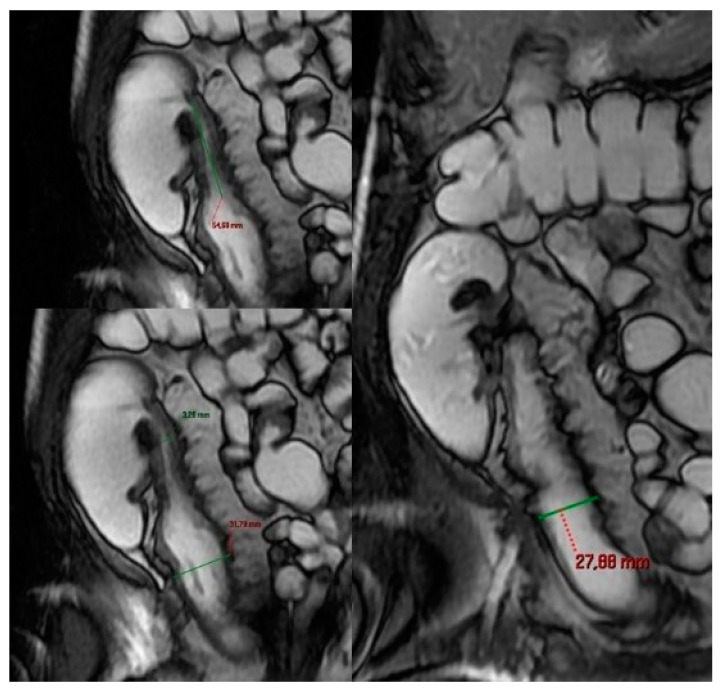
Non-functionally significant stenosis: there is an upstream bowel dilatation of less than 3 cm. The coronal FIESTA MRE image shows a perivalvular thick-walled ileal segment (approximately 6 cm long) with evident luminal narrowing and rigidity, not expandable with peristalsis but constantly fixed, albeit without upstream bowel dilation.

**Figure 9:**
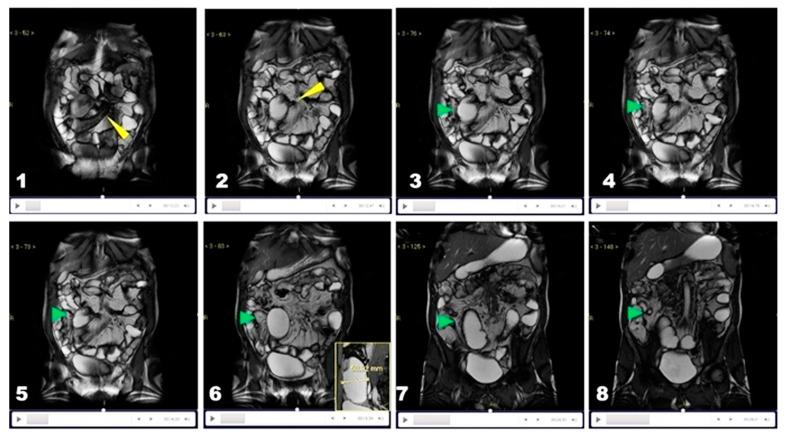
Condition of the “damned loop” in functional stenosis. We show the decomposition of the frames (**1**–**8**) of the Cine-MRE, which demonstrates how the peristaltic activity fails to overcome the stricture (yellow arrowheads in **1**,**2**), despite the attempts of the upstream loop to promote intestinal transit by contracting energetically, quickly, and vigorously as a species of a damned loop (green arrowheads).

**Figure 10:**
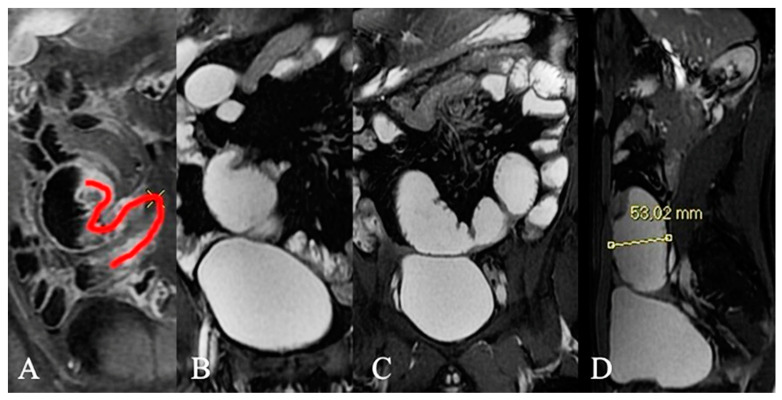
Very functionally significant stenosis: upstream bowel dilatation greater than 3 cm with constant and permanent ectasia of upstream bowel loops before high-grade bowel stenosis. Contrast-enhanced fat-suppressed T1-weighted image (**A**): The freehand red curve represents a relatively long, markedly stenotic segment of the ileum with marked enhancement, rigid and non-expandable. Permanent ectasia of upstream bowel loops before high-grade bowel stenosis in coronal (**B**,**C**) and sagittal (**D**) MRE in the same patient.

Finally, we must not neglect to look for direct and indirect signs of stenosis at the level of the pathological loops of the small intestine, as a stenosis can cause an upstream dilatation to either an inflammatory or fibrotic stricture. An upstream dilatation is significant when the small bowel diameter is >3 cm. In this scenario, cine imaging helps to distinguish between active inflammatory and fibrotic strictures and, more generally, between functional and non-functional stenosis where functional stenosis refers to unpassable stricture, in spite of a mighty peristaltic activity [44,46] (Figure 8, Figure 9 and Figure 10).

## 6. Conclusions

MRE is increasingly being used as a first-line imaging modality for the diagnosis of inflammatory disorders, and it has received clinical approval for the evaluation of gastrointestinal disorders other than Crohn’s disease.

When an abnormal small-bowel loop is recognized, it is indispensable to use a methodological approach, which underlies an overarching strategy that allows combining all pathological elements of the MRI to obtain an overall interpretation and decision.

Finally, we dutifully want to remind everyone that a reliable diagnosis is impossible if everything is entrusted to imaging without a deep integration with the clinical data.

## Figures and Tables

**Table 1:** Normal small bowel mean diameter (standard deviation) on MRE.

Jejunum	Proximal Ileum	Distal Ileum	Terminal Ileum
24.5 mm (S.D. = 4.2 mm)	19.5 mm (S.D. = 3.6 mm)	18.9 mm (S.D. = 4.2 mm)	18.7 mm (S.D. = 3.6 mm)

**Table 2:** Degree of thickening of the abnormal small bowel on MRE.

Mild	Moderate	Marked
3–4 mm	5–9 mm	≥10 mm

**Table 3:** Main bowel wall enhancement patterns.

Normal Enhancement	Normal Bowel Wall
Layered or stratification	two (double halo sign) or three (the target appearance); subtends a benign condition
homogeneous or hyperenhancement	global transmural high enhancement that uniformly compromises the entire bowel wall: present in active Crohn’s disease or in fibrosis, infiltration, ischemia, collagen deposition, or shock bowel
lower enhancement	Less avid mural enhancement is more typical of fibro-stenotic disease

## Data Availability

The data used during the current study are available from the corresponding author on a reasonable request.
